# Ranking buffel: Comparative risk and mitigation costs of key environmental and socio‐cultural threats in central Australia

**DOI:** 10.1002/ece3.6724

**Published:** 2020-10-26

**Authors:** John L. Read, Jennifer Firn, Anthony C. Grice, Rachel Murphy, Ellen Ryan‐Colton, Christine A. Schlesinger

**Affiliations:** ^1^ The University of Adelaide Adelaide SA Australia; ^2^ Queensland University of Technology Brisbane Qld Australia; ^3^ College of Science and Engineering James Cook University Townsville Qld Australia; ^4^ Charles Darwin University Darwin NT Australia; ^5^ Research Institute for the Environment and Livelihoods Charles Darwin University Alice Springs NT Australia

**Keywords:** Aboriginal welfare, Buffel grass, cost benefit analysis, extinction, invasive, refuges, weed control

## Abstract

Changed fire regimes and the introduction of rabbits, cats, foxes, and large exotic herbivores have driven widespread ecological catastrophe in Australian arid and semi‐arid zones, which encompass over two‐thirds of the continent. These threats have caused the highest global mammal extinction rates in the last 200 years, as well as significantly undermining social, economic, and cultural practices of Aboriginal peoples of this region. However, a new and potentially more serious threat is emerging. Buffel grass (*Cenchrus ciliaris* L.) is a globally significant invader now widespread across central Australia, but the threat this ecological transformer species poses to biodiversity, ecosystem function, and culture has received relatively little attention. Our analyses suggest threats from buffel grass in arid and semi‐arid areas of Australia are at least equivalent in magnitude to those posed by invasive animals and possibly higher, because unlike these more recognized threats, buffel has yet to occupy its potential distribution. Buffel infestation also increases the intensity and frequency of wildfires that affect biodiversity, cultural pursuits, and productivity. We compare the logistical and financial challenges of creating and maintaining areas free of buffel for the protection of biodiversity and cultural values, with the creation and maintenance of refuges from introduced mammals or from large‐scale fire in natural habitats. The scale and expense of projected buffel management costs highlight the urgent policy, research, and financing initiatives essential to safeguard threatened species, ecosystems, and cultural values of Aboriginal people in central Australia.

## Graphical abstract

This manuscript compares the risks to biodiversity and culture of central Australia posed by buffel grass and feral animals and fire. Our analyses show that the threat from buffel is at least as serious as other recognized threats but more expensive to manage. We advocate for more resources for research and control of buffel grass.

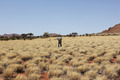

## 1 INTRODUCTION

Four contemporary ecological disruptions are widely recognized as dramatically and irreversibly recasting the ecology of Australia's arid and semi‐arid zones which cover over 70% of the continent, namely alteration of fire regimes as a consequence of displacement of Aboriginal peoples practicing traditional land management; overgrazing and soil compaction caused by the introduction of large exotic herbivores; predation of native wildlife by introduced red foxes (*Vulpes vulpes*) and domestic cats (*Felis catus*); and degradation of soils and vegetation by introduced European rabbits (*Oryctolagus cuniculus*) (Crabtree, Bird, & Bliege, 2019; Department of the Environment, 2008c; Landsberg, James, Morton, Muller, & Stol, 2003; McKenzie et al., 2007; Morton, 1990; Woinarski, Burbidge, & Harrison, 2014). Collectively, and often in concert, these four threatening processes have been largely responsible for the highest contemporary mammal extinction rates in the world and a bourgeoning inventory of threatened species (Kearney et al., 2018). They are also responsible for broadscale losses in productivity and an erosion of traditional Aboriginal cultural values in central Australia (Woinarski, Burbidge, & Harrison, 2015). To address these threats, Australian governments, nongovernment organizations, and private landholders invest tens of millions of dollars annually in feral animal control programs (Saunders, Coman, Kinnear, & Braysher, 1995; Woinarski, Legge, & Dickman, 2019) including the creation and maintenance of fenced predator‐proof sanctuaries, implementation of fire management strategies, and support for “Working on Country” initiatives to provide options for Aboriginal people to re‐engage in land management practices (Garnett, Latch, Lindenmayer, & Woinarski, 2018; Legge et al., 2018).

In recent decades, a fifth major threat to Australian arid and semi‐arid zone ecosystems has emerged and it is considered by many ecologists as “the single biggest invasive species threat to biodiversity across the entire Australian arid zone” (Biosecurity SA, 2019). Buffel grass *(Cenchrus ciliaris* L., hereafter buffel) is also introduced and invasive in mainland USA, Mexico, parts of Central and South America (Lyons, Maldonado‐Leal, & Owen, 2014; Marshall, Lewis, & Ostendorf, 2012; Williams & Baruch, 2000), Papua New Guinea, and Pacific Islands including Fiji, Tahiti, and the Hawaiian Islands (PIER, 2014). In Australia, many varieties of buffel have been deliberately introduced from 35 countries by various agencies (Hall, 2000) as hardy cattle forage and a dust suppressant. Plantings have occurred mostly since the late 1950s (Paull & Lee, 1978) and are continuing in some areas (Friedel, Puckey, O'Malley, Waycott, & Smyth, 2006), despite buffel now being internationally recognized as causing broadscale and multilayered negative impacts on ecosystem processes, biodiversity, infrastructure, and human safety (e.g., Franklin & Molina‐freaner, 2010; Godfree et al., 2017; McDonald & McPherson, 2013; Olsson, Betancourt, McClaran, & Marsh, 2012; Schlesinger, White, & Muldoon, 2013; Woinarski, 2004).

Buffel is considered a transformer species because of its ability to alter invaded environments (Grice, 2006), by forming dense swards and increasing fire connectivity in areas that were previously more sparsely or patchily vegetated (Clarke, Latz, & Albrecht, 2005), thereby enabling increased frequency, intensity, and extent of fire (Butler & Fairfax, 2003; Edwards, Allan, et al., 2008; Edwards, Zeng, Saalfeld, Vaarzon‐Morel, & McGegor, 2008; Miller, Friedel, Adam, & Chewings, 2010). For example, in invaded riparian habitat in central Australia, cover of buffel was close to prefire levels sixteen months after fire (Schlesinger et al., 2013); these invaded areas burnt twice within a decade, despite concentrated efforts to protect the area with fire breaks (CAS pers. obs.). With the exception of spinifex (*Triodia* spp.) grasslands, the major native vegetation types of inland Australia historically experienced fire only following periods of above average rainfall over two or three consecutive seasons (Edwards, Allan, et al., 2008; Edwards, Zeng, et al., 2008), conditions that have tended to occur only at multidecadal intervals. Long‐lived trees and shrubs are damaged and killed by repeated, intense buffel‐fueled fires (Marshall et al., 2012; Schlesinger et al., 2013). Obligate seeder species cannot persist if fire return intervals are shorter than their time to reach reproductive maturity (Edwards, Allan, et al., 2008; Edwards, Zeng, et al., 2008) with the inevitable consequence that diverse plant communities will be replaced by buffel monocultures. Increased frequency and intensity of fires fueled by buffel also threaten important cultural sites, and dense buffel monocultures restrict traditional bush tucker and hunting activities (Biosecurity SA, 2019; UKTNP, 2009).

Whereas other serious weeds of central Australia, like *Tamarix aphylla* and *Parkinsonia aculeata,* are outcompeted by established natural vegetation or are largely restricted to agricultural or disturbed areas with enhanced water or nutrient availability (Grice & Martin, 2006), buffel is an aggressive invader of many Australian arid and semi‐arid zone habitats (Fensham, Wang, & Kilgour, 2015; Firn et al., 2015; van Klinken & Friedel, 2018). Buffel directly suppresses (Abella, Chiquoine, & Backer, 2012; Eilts & Huxman, 2013) and threatens the persistence of many native plants (Clarke et al., 2005; Edwards, Schlesinger, Ooi, French, & Gooden, 2019; Eyre, Wang, Venz, Chilcott, & Whish, 2009; Fairfax & Fensham, 2000; Friedel et al., 2006) including threatened species (Griffin, 1993; Jackson, 2005). Changes in vegetation composition and structure following buffel invasion also affect species and assemblages of fauna (Bonney, Andersen, & Schlesinger, 2017; Pavey & Nano, 2009; Read & Ward, 2011; Smyth, Friedel, & O’Malley, 2009; Williams, Mulligan, Erskine, & Plowman, 2012; Schlesinger, Kaestli, Christian & Muldoon, 2020; Young & Schlesinger, 2014) and may increase the risk of extinctions. There is substantial evidence that buffel establishment in arid and semi‐arid communities has negatively affected native biodiversity (Bonney et al., 2017; Griffin, 1993; Marshall et al., 2012; Schlesinger, et al., 2020), even at comparatively low cover levels (Eyre et al., 2009; Friedel et al., 2006) though the mechanisms driving these impacts are not yet well understood. As a consequence of these risks, and the much wider geographical range of buffel compared with most other invasive species, buffel has been recognized as Australia's most serious invasive grass and one of the key threats to biodiversity in many arid regions, including the Pilbara, Kimberley, Lake Eyre Basin, Brigalow Belt, and much of northern South Australia (Biosecurity SA, 2019; Carwardine et al., 2014; Chades et al., 2014; Firn et al., 2015; Ponce‐Reyes et al., 2016).

Eradicating established animal pests at continental scales has proved impossible (Bomford & O'Brien, 1995) and, similarly, complete removal of widespread invasive plant species from mainland Australia is unlikely feasible or, in the case of buffel, desirable. Buffel is currently estimated to underpin approximately 44% of the $17 billion beef cattle enterprises in Australia (G. Campbell personal communication), and its eradication would be detrimental to the pastoral industry (Friedel, Grice, Marshall, & van Klinken, 2011; Grice, Friedel, Marshal, & van Klinken, 2012). The economic benefits of buffel—or of any of the other introduced species we compare it with—are not the focus of our research but we acknowledge that they are substantial. Conversely, we also note that, in some situations, assisted or accidental spread of buffel into pastoral regions can, paradoxically, result in loss of pastoral production through elevated oxalate concentrations sufficient to cause calcium deficiency (Cheeke, 1995) and acute oxalate poisoning (Offord, 2006; Thomas, 2004) in livestock and replacement of more productive perennial and ephemeral pastures and drought forage (NRSA Arid Lands, 2017). The most serious economic threat for the pastoral industry caused by buffel pasture may be drawdown of nitrogen and other nutrients with an associated decline in cattle live‐weight gain (Graham, 2000; Puckey & Albrecht, 2004) and a halving of carrying capacity in buffel pastures after 10–20 years, predicted to cost the cattle industry over $17 billion over the next 30 years (Peck et al., 2011). Furthermore, an undiagnosed and as yet untreatable buffel dieback potentially threatens the future viability of buffel pastures (Makiela & Harrower, 2008). Nevertheless, the extent of these issues is not well established, and some are region‐specific. We do not suggest they significantly detract from the current economic benefits of increased productivity associated with buffel pasture in most regions. The increased risks to infrastructure, drought refuge, livestock, and humans associated with buffel‐fueled fire are probably of most concern to pastoralists. There has been consensus among stakeholders, including pastoralists, that buffel is undesirable in conservation reserves and should be controlled outside pastoral areas (Friedel et al., 2011).

Widespread concern about the negative impacts of buffel has been the impetus for appeals for a comprehensive, coordinated, and strategic national approach to its management (Reynolds, 2012), but such national policies and action plans have not eventuated. Buffel was gazetted as a declared weed in South Australia in 2015, is listed as a “significant threat” in the Alice Springs Regional Weed Management Plan 2013–2018 (DLRM, 2013), and is included in the list of key threatening processes under the New South Wales *Threatened Species Conservation Act 1995*, Invasion of native plant communities by exotic perennial grasses. Other high biomass non‐native grasses of northern Australia have been identified as Key Threatening Processes under the Commonwealth Environment Protection and Biodiversity Conservation Act 1999 (Anon., undated). Yet buffel has not been listed as a Weed of National Significance or listed under Federal legislation, despite an application in 2013, due largely to its value as a pasture grass for dry tropical regions (Friedel et al., 2011; Grice et al., 2012; Marshall et al., 2012). Despite prioritization studies clearly identifying buffel as a high‐priority invasive species in Queensland (Firn et al., 2015; Ponce‐Reyes et al., 2016), conflicting valuations by pastoralists versus environmental interests may account for the decision not to include buffel in an inventory of environmental weeds in Queensland (Osunkova et al., 2019). Much of the past debate around buffel has focussed on agro‐economic benefits versus environmental costs (Godfree et al., 2017), without considering the cultural and socioeconomic impacts on stakeholders other than pastoralists, which should also be accounted for in management decisions (Crowley, Hinchliffe, & McDonald, 2017; Hoagland & Jin, 2006), potentially engaging the public in ways that environmental impacts alone do not (Genovesi et al. 2014). Particularly, impacts on Aboriginal people, the majority of residents in remote arid Australia and traditional custodians of the land, have been almost completely ignored. In the absence of a coordinated national strategy or research, management of buffel remains largely the responsibility of local jurisdictions and, more commonly, individual land managers who have inherited the challenge of attempting to protect key refuge areas or other assets.

## 2 THE ROLE OF REFUGES IN MANAGING THREATS

We regard refuges as areas where threatening processes are markedly reduced by physical barriers, intensive management, or spatial buffers provided by broadscale management. Although we recognize that shifting refuges are often more ecologically viable in arid areas (Reside et al., 2019), sanctuaries provide a form of refuge by excluding threatening processes from intensively managed (typically fenced) areas. Sanctuaries may be both cost‐effective and integral to retaining sensitive species, while wide‐ranging remedies, including biological control, are developed (e.g., Carwardine et al., 2014; Hayward, Moseby, & Read, 2014). Although multiple threatening processes are managed within sanctuaries and benefits extend to a wide range of fauna and flora (Moseby, Hill, & Read, 2009; Moseby, McGregor, Hill, & Read, 2019; Munro, Moseby, & Read, 2009), the protection of endangered species through the exclusion of introduced predators is usually the primary reason for their establishment. As of early 2018, there were 19 effective feral cat‐ and fox‐proof sanctuaries (total area 35,000 ha) on the Australian mainland and they have become the cornerstone of efforts over the past three decades to protect 49 populations of threatened Australian fauna from predation. The establishment of an additional 91,400 ha of predator‐proof sanctuaries is imminent (Legge et al., 2018). In arid Australia, the larger sanctuaries for threatened mammals, including Scotia, Arid Recovery, and Newhaven, supporting viable populations of threatened herbivores and omnivores, are approximately 1,000 ha. We consider 10,000 ha also provides sufficient area for localized traditional bush food gathering and small game hunting, based on maximum walking distances of 8–12 km for hunting and gathering trips for Aboriginal people in arid Australia (Walsh, 2008).

For threatening processes other than introduced predators, broadscale management is often more feasible and more appropriate than creating sanctuaries. For example, a $19 million control program removed 160,000 feral camels (*Camelus dromedarius*) from central Australia between 2009 and 2013 (Ninti One Limited, 2013). Suppression of foxes within unfenced refuges through long‐term widespread baiting has provided demonstrable benefits for some threatened species, with reversals in decline of vulnerable wallaby species recorded (e.g., Brandle, Mooney, & de Prue, 2019; Burrows et al., 2003; Kinnear, Onus, & Sumner, 1998). Feral cat management is the focus of a National Threat Abatement Plan with a five‐year forecast cost of $19 million, excluding education, training, and individual island eradications that are costed at between $18,000 and $44 million each (Commonwealth of Australia, 2015). Concerted efforts are also being made to manage fire in more appropriate ways, including as a key focus of the Ten Deserts and Central Land Council Indigenous Ranger Programs (e.g., CLC, 2015), targeting areas with particularly fire‐sensitive biota, even though information is still lacking about the effectiveness of these strategies at large spatial scales (Nano, Clarke, & Pavey, 2012). Successful releases of biological control agents including *Cactoblastis* for control of the invasive prickly pear (*Opuntia* spp.) (Mann, 1970) and rabbit‐specific viruses for control of European rabbits have reduced populations of invasive species and facilitated widespread recovery of threatened species and ecosystems in arid and semi‐arid Australia (Mutze et al., 2014; Pedler et al., 2016).

Likewise, efficient management of buffel grass requires landscape‐scale approaches. Potential endemic biocontrol agents for buffel include the Australian moth caterpillar *Mampava rhodoneura* that damages buffel seed heads and feeds on seeds (Friedel et al., 2006). Buffel blight, caused by the fungal pathogen *Pyricularia grisea*, and ergot (*Claviceps* spp.) affecting seed production (Perrott, 2000) are already found in Australia. Dieback of buffel has been documented in some Queensland pastures (Makiela, 2008; Makiela & Harrower, 2008), with recent evidence suggesting the mealybug *Heliococcus summervillei* may be the causative agent. However, no policy objectives or dedicated research into these or other potential biocontrols for buffel have been instigated. Indeed, the focus of current research is to minimize the effects of endemic buffel biocontrols. Buffel management is therefore largely restricted to attempts to remove it by mechanical and chemical means from priority areas of the conservation and public estate, and Indigenous Protected Areas, to protect biodiversity and cultural practices (Bardsley & Wiseman, 2012; Dixon, Dixon, & Barrett, 2001; Friedel, Marshall, van Klinken, & Grice, 2008). However, such management is only realistic at small scales and requires continual inputs. A landscape‐scale approach is required to substantially reduce threats to environmental values and may be feasible, especially if focussed on areas not yet heavily invaded and on controlling spread to areas of high value for biodiversity and cultural practice by Aboriginal peoples.

Although patchy buffel in recently invaded, otherwise natural environments is unlikely to significantly erode biodiversity or cultural values, buffel often dominates vegetation communities within decades of invasion. Therefore, the most effective contemporary buffel management focusses on preventing buffel establishment from parts of the Great Victoria Desert and recently invaded southern regions of South Australia (Biosecurity SA, 2019). However, long‐term efficacy of such exclusion zones, which do not exist in other States and Territories of Australia, has not been demonstrated. In the absence of biological limitations, early intervention for prevention and eradication of pest plants and animals is typically cheaper and more effective than ongoing control (DEWR, 2006; Genovesi, 2011). Creating and maintaining landscape‐scale buffel‐free sanctuaries appears to be the most viable mechanism available to conserve biodiversity and enable continuation of cultural practices of Aboriginal peoples, including hunting and bush tucker collection. However, there are no current or proposed landscape‐scale sanctuaries within buffel‐invaded environments, nor is there legislation, policy, or funding earmarked for such efforts. For instance, broadscale buffel control at the World Heritage Listed Uluru‐Kata Tjuta National Park remains an insurmountable challenge, and current management is now restricted to targeted priority sites with particularly high identified cultural and biodiversity values. These sites include an enclosure for a captive population of mala (*Lagorchestes hirsutus*) and waterholes and rock art sites around the base of Uluru (M. Misso, Parks Australia personal communication).

Previous research has focussed on comparing potential costs of buffel with economic benefits (Friedel et al., 2006a). However, our review focuses on assessing the threat buffel poses to biodiversity and cultural values, especially of Aboriginal peoples, and the estimated costs of managing that threat compared with other major, well‐recognized threats: rabbits; large exotic herbivores; cats and foxes; and large‐scale, intense fires. Specifically, we estimate where buffel ranks relative to other threats in terms of 1) biodiversity risks, 2) cultural risks, and 3) funds needed to create a 10,000‐ha refuge from the threat. We then use our cost and risk data to outline new policy, research, and funding priorities for improved and coordinated buffel control.

## 3 METHODS

### 3.1 Assessment of biodiversity risks

Our assessment of the relative scale and severity of biodiversity risks posed by the five main threatening processes in arid and semi‐arid Australia was constrained by available information on risks to threatened species. The relatively recent recognition of the threats of buffel and the concomitant under‐resourcing of research identifying taxa threatened by buffel incursion limit comprehension of the range and scale of at‐risk species. Here, we acknowledge this deficiency and use a combination of management plans, State and Federal threatening process abatement plans, recovery plans for threatened species, and expert knowledge to conservatively estimate the risk to threatened species from buffel. Expert knowledge was synthesized by the authors (from Universities in three jurisdictions) drawing on their own environmental monitoring and research experience and using broadscale community and expert opinions including those from multistakeholder workshops ranking threatening processes in rangelands (Carwardine et al., 2014; Firn et al., 2015). Lists of fire‐sensitive communities and species were derived from management plans for Indigenous Protected Areas, and we assume that buffel invasion will increase fire threat to this biota. National action plans for the management of rabbits, cats, foxes, and camels informed our risk assessments of these threatening processes. Camels were used as a representative of large ungulates because more data are available on the threats posed by camels and their management costs compared with other species.

### 3.2 Assessment of cultural risks

To rank the relative risk and socio‐cultural impacts of different threats for Aboriginal peoples living in remote Australia, we used a customized version of the Socio‐Economic Impact Classification for Alien Taxa (SEICAT) (Bacher, Blackburn, & Ess, 2018) that measures changes in the realized activities of people. This classification system accounts for the expected lack of knowledge of the impact on many components of cultural well‐being by using maximum known impact and by focussing on components that are particularly affected or important, rather than assessing an exhaustive list of possible impacts (Bacher et al., 2018). We focussed specifically on the impacts on socio‐cultural values of Aboriginal people in central Australia given they are the traditional custodians of the land and are likely to experience a multitude of impacts due to their close connection to the environment.

Given the limited peer‐reviewed or gray literature available to draw on, we felt it was important to supplement these sources of information with elicitation of expert opinion, which is commonly used for risk analysis in ecology and natural resource management (McBride et al., 2012; Speirs‐Bridge et al., 2010). Five of the six co‐authors (JLR, CAS, JF, AG, and ER) independently assessed the magnitude of current and future impact of each of the six threats on the identified cultural values guided by SEICAT protocols, and based on the available sources of information and their opinion, following a modified Delphi process for elicitation of expert opinion (McBride et al., 2012). The authors that contributed to the assessment have worked closely with Aboriginal people in diverse regions of inland Australia for a minimum of four and maximum of 25 years, during which much anecdotal information has been shared by Aboriginal informants, colleagues, senior knowledge holders, and other community members, as acknowledged at the end of this paper. First, we listed cultural values known to be impacted by at least one of the six main threats under consideration (foxes and cats separated) using peer‐reviewed and gray literature sources including written articles and reports, interview transcripts, and multimedia from over 10 Aboriginal language groups, across central Australia including north to the Tanami Desert, south to Lake Eyre Basin, and west to Western Australia (e.g., Batty & Walsh, 2012; Vaarzon‐Morel, 2010; West, Nangala, Wright, & Crossing, 2018). Building from this, we constructed an assessment table that included values that were representative of the breadth of social and cultural values potentially threatened, including material assets, nonmaterial assets, regulation of the environment (sensu Diaz et al., 2018), and human well‐being (Bacher et al., 2018). The individual assessments were tallied using the median score if the authors’ rankings were within one category of the median. Where opinions diverged more widely (which occurred in *n* = 16, from a total of 78 threat/value combinations), authors were shown the other assessors’ scores and given the chance to reassess their own scores (McBride et al., 2012). “No consensus” was subsequently recorded for only one threat/value combination after the reassessment.

We do not consider ourselves experts on Aboriginal cultural and social perspectives—instead, we are expert ecologists who have worked with and actively listened to Aboriginal perspectives on ecological issues over prolonged periods, albeit often informally. We do not presume to speak for Aboriginal people and emphasize that our results represent our opinions, informed by evidence that is available to us and do not provide authoritative decisions about cultural impacts for Aboriginal people. We acknowledge especially that information about deeper cultural values is not always shared, which makes the assessment of nonmaterial aspects and human well‐being even more difficult. However, the SEICAT scheme, which focusses on the change in the size, location, or type of people's activities, provided a consistent measure from which to base our assessments of the relative threat to the identified values from each threatening process. We also recognize that for Aboriginal people in central Australia, the values of land, nature, law, spirituality, and people are connected as “country” and cannot easily be categorized (Pawu‐kurlpurlurnu, Holmes, & Box, 2008), and therefore, the impacts listed here are interconnected and context‐dependent and will vary across the region, although we tried to apply a regional view in our assessments rather than focussing on individual communities.

We follow the approach of Bacher et al. (2018) of assessing the magnitude only for negative cultural influences, which have previously not been considered, also consistent with the focus of our paper, and have concentrated our review on the substantial areas of inland Australia where residence and day‐to‐day maintenance of cultural practice by Aboriginal people, tourism, and biodiversity conservation are the principal land uses. We have already acknowledged that pastoral production supported by buffel has cultural and economic benefits for some pastoralists, and this includes some Aboriginal pastoralists, who have deliberately or inadvertently replaced native pastures with buffel. Aside from pastoral production, and associated cultural practice, we acknowledge that some of the threats may have positive impacts on socio‐cultural values such as dust suppression (buffel) or providing a new food source (rabbits, cats) and these have been noted but not categorized in the SEICAT scheme.

### 3.3 Cost of creating refuges

Costing the creation and maintenance of refuges from the five key threatening processes is complicated by the often‐interactive nature of these threats. For example, fox and cat populations are typically higher and more difficult to control in areas of high rabbit density (Read & Bowen, 2001), buffel is typically suppressed by heavy grazing pressure from exotic herbivores and buffel density, and fire frequency and intensity are highly synergistic (Butler & Fairfax, 2003). Such interactions were factored into our costings for each threatening process. For example, increased fire management costs were budgeted for areas with a buffel infestation.

We applied a consistent and pragmatic approach to determining costs of creating and maintaining refuge areas for different threats. Hypothetical refuges were located in habitats at risk from the threatening process but not where habitat features or degradation made establishment of refuges impractical. For example, fencing and maintenance costs make rabbit‐ or predator‐proof exclosures impractical in areas with watercourses or steep rocky hills, so fencing costs assumed construction on relatively flat terrain, remote from rocky hills or erosion features. Likewise, it would not be practical to select an area for refuge creation where buffel had already formed a monoculture. Rather, we assumed that refuges would be created near the invading front of buffel, where future dense infestations were inevitable without intensive management.

Costs were estimated for creating and maintaining a 10,000‐ha refuge from each threatening process for 20 years, recognizing that it would often be logical to manage multiple threats within the same refuge. Because fencing is typically a prerequisite to ensure mainland sanctuaries are free from large exotic herbivores, rabbits, foxes, and cats, the capital, operational, and management costs of different types of fencing and eradication programs determined the main costs of refuges from these threats. While representing less than 1% of projected costs of sanctuary management, exclusion of ungulates that can spread buffel in their hoofs, hide, or dung is considered integral to creating and maintaining a sanctuary from buffel and was included in the costing for a buffel‐free sanctuary. Conversely, refuges from fire were assumed to not require fencing and most costs were associated with ongoing management. Case studies of successful and unsuccessful buffel and fire management were used to estimate 20‐year costs of creating a 10,000‐ha sanctuary.

## 4 RESULTS

### 4.1 Biodiversity risk profile for different threats

#### 4.1.1 Feral predators

Feral domestic cats and European red foxes represent a threat to central Australian wildlife through predation and transmission of disease. These non‐native predators have been largely responsible for the extinction of 22 Australian native mammals and are currently listed as a threat to 50 species of birds, 82 species of mammals, 19 species of reptiles, and five species of amphibians (Department of the Environment, 2008a, 2008b). Feral cats alone are recognized as a threat to 74 mammal species and subspecies (Woinarski et al., 2014). In addition, 40 bird, 21 reptile, and four amphibian species listed as threatened under the Environment Protection and Biological Conservation 1999 (EPBC) Act are considered threatened by feral cats (Woinarski et al., 2014, Table 1).

**TABLE 1:** Summary statistics for Environment Protection and Biodiversity Conservation Act 1999 (EPCB)‐listed taxa and expert opinion of authors on scale of threat (source references in text) * inappropriate fire regimes are listed as a potential threat for most listed plant species within the Australian semi‐arid and arid zones but with little evidence of direct threats to any particular species (J. Silcock personal communication) ** aquatic invertebrates are mentioned as threatened but species inventory not provided

Threat	No. of EPBC‐listed plant species impacted	No. of EPBC‐listed terrestrial animal species impacted	Likelihood of significant underestimate of no. of threatened species	Potential to increase geographic range of threat	Inefficacy of existing control	Relative conservation risk rating
Camels	16	**	Low	Low	Mod	Low
Rabbits	121	35	Low	Low	Low	Mod
Inappropriate fire regimes	*	44	Mod	Low	Mod	Mod
Foxes and cats	0	139	Low	Low	Mod	High
Buffel	6	21	High	High	High	High

#### 4.1.2 Large exotic herbivores

Large exotic herbivores threaten some plant species through direct herbivory and trampling and place aquatic biota at risk through fouling or drinking remote waterbodies (Brim Box, McBurnie, et al., 2016). They also reduce wildlife visitation to waterholes (Brim Box et al., 2019). Sixteen plant species, including one vulnerable species (*Santalum acuminatum*), are considered highly vulnerable to local extinction from camel grazing in central Australia (Edwards, Allan, et al., 2008; Edwards, Zeng, et al., 2008, Table 1), although there is no quantitative evidence of increased adult mortality and declines in palatable species (Brim Box, Nano, et al., 2016).

#### 4.1.3 Rabbits

European rabbits affect native flora and fauna by grazing and preventing regeneration. They compete with native herbivores for food and their digging and browsing lead to a loss of vegetation cover, slope instability, and soil erosion (Department of the Environment, 2008c). Nineteen threatened (EPBC‐listed) birds, 13 threatened mammals, two threatened reptiles, one threatened insect, and 121 threatened plants are considered at risk from rabbits (Department of the Environment, 2008c). Although the reprieve may not be permanent, dramatic reduction in rabbit numbers since the introduction of rabbit hemorrhagic disease in the mid‐1990s reduced the biodiversity threat and significantly improved the conservation prospects for several threatened animals in central Australia for at least 20 years (Pedler et al., 2016).

#### 4.1.4 Changed fire regimes

Many plants and animals of central Australian ecosystems are not well‐adapted to the changed frequency, intensity, and extensive nature of contemporary fire regimes. Where little cover remains following a fire, wildlife are more exposed to predation (Körtner, Pavey, & Geiser, 2007; Letnic & Dickman, 2005), especially by feral cats (McGregor, Legge, Jones, & Johnson, 2014; Southgate, Paltridge, Masters, & Ostendorf, 2007). EPBC‐listed species threatened by inappropriate fire regimes include 51 birds, 69 mammals, 20 reptiles, six fish, nine frogs, and 25 other species (Commonwealth of Australia, 2015). Many vertebrates, including a suite of hollow‐dependent birds, mammals, and reptiles, are directly threatened by increased fire intensity and frequency in woodland habitats (Neave et al., 2004). Fauna species that require mature spinifex (*Triodia* spp. and *Neurachne* spp.) (e.g., striated grasswrens (*Amytornis striatus),* night parrots (*Pezoporus occidentalis*), and sandhill dunnarts (*Sminthopsis psammophila*)) or old‐growth mallee (e.g., malleefowl *Leipoa ocellata*) are considered especially sensitive to inappropriate fire regimes.

#### 4.1.5 Buffel

Through direct competition and fueling more frequent and intense fires, buffel is considered to threaten at least 12 EPBC‐listed mammals, five birds, three reptiles, one insect, and six plant species. However, there has been considerably less research to identify the effects of buffel on fauna, compared with the other threats we have considered. Although beyond the scope of this study, a Google Scholar search, which did not account for geographic range or land use, suggests that rabbits, cats, and foxes have each been included in 8–13 times the number of research articles, whereas livestock and fire have each been considered in over 150 times the quantity of research on biodiversity impacts than buffel. As a consequence and due to the relatively recent nature of the threat in many areas, the inventory of threatened and more abundant biota, ecosystem structure, and function vulnerable to buffel invasion is likely to be far greater than has been documented (KTP, 2012, Table 1). For example, when the inventory of fauna threatened by buffel was collated in 2012 for the Key Threatening Process Nomination (KTP, 2012), the night parrot was not included despite subsequent research identifying buffel encroachment as a serious threat to this endangered bird (Murphy et al., 2017). Likewise, in the long term, many other fauna endemic to *Triodia* habitats throughout Australia, including the world's most diverse reptile communities (Pianka, 1986), are also potentially threatened by buffel which has now replaced the distinctive *Triodia* hummocks with grass tussocks over extensive areas in some regions. Although buffel seed is being included in the diet of some native granivores, experimental trials suggest native grasses are preferred (Young & Schlesinger, 2018); buffel is almost entirely replacing native grasses in invaded areas and the effects on native granivores are unknown.

### 4.2 Cultural risk profile for different threats

Despite its relevance to prioritizing management actions, we were able to find few published Aboriginal perspectives about the relative risks to their social and cultural values caused by the six threatening processes. The exception is feral camels where impacts on natural and cultural resources, especially waterholes, have been noted to be of major concern (Vaarzon‐Morel, 2010) and the impact of large‐scale wildfires on cultural sites (Gabrys & Vaarzon‐Morel, 2009). Evidence of the negative effects of buffel on cultural values for remote Aboriginal communities of inland Australia was sourced from multimedia documentaries and videos (Batty & Walsh, 2012; Courtney, 2015; Frazer, 2012; NG Media, 2017; Ninti Media & PIRSA, 2016) where Aboriginal people discussed impacts on bush food collection and hunting, how access to traditional lands changes when buffel dominates the landscape, and cascading negative effects on cultural transmission to younger generations and maintaining options for cultural practice. For example, Aboriginal people of central Australia are now reluctant or unable to conduct traditional fire management due to the increased intensity of buffel fires and quick recovery of buffel postfire (Bardsley & Wiseman, 2012; Read et al., 2018). Aboriginal people recognized the impact of cats on prey animals which are valued as totem species or more generally for their contribution to biodiversity (West et al., 2018). Rabbits and cats were also valued positively as food resources, whereas dense infestations of buffel, referred to as “devil grass” by some senior Aboriginal women, were most often highlighted in sources as being detrimental to culture. These differences in expressed concerns about threats may relate to the time since impacts were first realized and whether management is in place: Buffel grass and camels are very much viewed as a current threats, whereas rabbits, cats, and foxes, which invaded the region up to 100 years ago, seemed to be of less concern, except for people who had experience working on feral animal management programs. People have become accustomed to the presence of feral animals in the landscape over generations, and in the case of rabbits, impacts have diminished in severity significantly since the widespread biocontrol measures of the past few decades. With the exception of camels, which pose a direct threat to infrastructure and can cause direct injury to humans, the impacts of feral animals are also less obvious compared with fire and buffel invasion. Multimedia sources with recorded interviews with Aboriginal people, and the overall ranking of threats to cultural values by the authors (Table 2), suggest that buffel poses the greatest contemporary threat to the social and cultural values of Aboriginal people living on country in inland Australia compared with the other threats we considered (Biosecurity SA, 2019, Table 2).

**TABLE 2:** Socio‐Economic Impact Classification for Alien Taxa conducted by authors for six main threats, with the assessment of major and massive impacts highlighted in bold (adapted from Bacher et al., 2018)

Values to Aboriginal people in central Australia	Large wildfires	Camels	Rabbits	Foxes	Cats	Buffel
*Material assets including resources, employment, and income*						
Bush food, bush medicines, and materials (plants)	MO^a^	MO^a^	MN	MC	MC	**MV** ^a^
Hunting (animals)	MN^a^	MN^a^	MC +	MO	MO+^a^	**MR** ^a^
Tourism	MO	MC+^a^	MC	MC	MC	MO
Income generation from livestock	MO	MN+^a^	MN	MC	MC	MC +
Other economic impacts (including damage to infrastructure)	MO^a^	MO^a^	MN	MC	MC	MO^a^
*Nonmaterial assets including cultural values and good quality of life*						
Personal and community safety	MO^a^	MN^a^	MC	MC	MC	MO^a^
Culturally significant animal and plant species (including totem species and in Aboriginal law)	MN^a^	MN^a^	MN	**MR**	**MR** ^a^	**MR**
Damage to cultural sites	MO^a^	**MR** ^a^	MC	MC	MC	MO^a^
Intrinsic and aesthetic environmental values (flowers, birds, overall biodiversity)	MN^a^	MN^a^	MN	MN	MO^a^	**MR** ^a^
Ability to walk country including learning on country with elders	MN	MO^a^	MC	MC	MC	**MR** ^a^
Overall quality of life (health, happiness, distress)	MO	MN^a^	MC	MN	MN	**MR** ^a^
*Regulating the environment*						
Dust impacts	MN	MN	No consensus	MC	MC	MC+^a^
Water quality	MN^a^	**MR** ^a^	MC	MC	MC	MC
Maximum magnitude of impact	Moderate	Major	Minor	Major	Major	Massive

Note

Minimal concern (MC): No deleterious impacts reported or known despite relevant studies suggesting potential for impact on human well‐being. Minor (MN): Negative effect on peoples’ well‐being, making it difficult for people to participate in some normal activities. For overall ratings, individual people suffer in at least one constituent of well‐being (i.e., security; material and nonmaterial assets; health; social, spiritual, and cultural relations). Moderate (MO): Negative effects on well‐being leading to changes in activity size, fewer people participating in an activity, or moving activity to unaffected regions. Major (MR): Local disappearance of an activity from all or part of the affected area. Collapse of the specific social activity within the community but change likely reversible within a decade if alien taxon is controlled. Massive (MV): Permanent and irreversible disappearance of an activity for at least a decade, due to fundamental structural changes of socioeconomic community or environmental conditions (“regime shift”). The symbol (+) acknowledges a positive impact in addition to any negative impact but the magnitude is not rated within this scheme. No consensus indicates that expert opinion varied substantially even after the modified Delphi process of consensus building.

^a^ Indicates where peer‐reviewed, and secondary source material was available to help inform opinion.

### 4.3 Estimated refuge costs

#### 4.3.1 Large exotic herbivores (20‐year cost for 10,000 ha ca $280,000)

Large exotic herbivores, including camels, horses, donkeys, and cattle, can typically be excluded from 10,000‐ha paddocks by three‐line barbed wire fencing, especially if water points are remote from those fences. Construction of these standard stock fences typically costs approximately $2,000 per km in materials and labor and requires little ongoing maintenance, especially if not constructed over watercourses. The cost of erecting a 10,000‐ha camel‐proof exclosure with 40 km of fencing would therefore be approximately $80,000.

Costs for mustering, trapping, or shooting large exotic herbivores from a 10,000‐ha sanctuary and ongoing intermittent fence repairs would likely amount to less than $10,000 per year if skilled workers were available nearby, or $200,000 over a 20‐year timeframe. Alternatively, an unfenced 10,000‐ha sanctuary could be largely maintained by aerially shooting an indicative 200 large exotic herbivores a year from a 30,000‐ha buffer area each year at a cost of ca $37,000 per year (M. Zabek personal communication) or $740,000 over 20 years. Although these costs could potentially be incentivized by tax credits for Aboriginal employment, they exceed indicative exclusion fencing costs and hence have been disregarded from our modeling.

#### 4.3.2 Cats and foxes (20‐year cost for 10,000 ha ca $2,130,000); foxes alone (20‐year cost for 10,000 ha ca $1,076,000)

In addition to sometimes considerable approvals and planning costs (Pedler et al., 2018), cat‐ and fox‐proof fences typically cost between $31,000 and $40,000 per km (Ireland et al., 2018; R. Pedler (Wild Deserts) pers. comm. 2018; Woinarski et al., 2019) to construct depending on their size and location. Assuming $35,000 per km for materials and construction, a 40km cat‐ and fox‐proof fence enclosing 10,000 ha would cost approximately $1.4 million. Such a fence would also exclude large exotic herbivores, although in areas where these are in high densities, supplementary electric wires or more closely spaced posts may be required to prevent damage to the floppy top barrier of the fence.

Annual fence maintenance costs for similar‐sized reserves range from $13,000 (Hayward et al., 2014) to $25,000 (Moseby & Read, 2006) suggesting a 20‐year maintenance cost of $500,000 for a 10,000‐ha sanctuary. The foot netting that is essential to exclude feral predators typically requires replacement after ca 20 years, which could add an additional $100,000 to the 20‐year maintenance budget.

Experience from Arid Recovery, Scotia, and the Warru Pintji sanctuaries suggests that use of floppy top fencing (that permits cats and foxes to climb out but not in) greatly assists with cat and fox eradication. One trained worker valued at $7,000 per month would be expected to eradicate these feral predators from relatively open arid habitats with 6 months of intensive effort costing ca $50,000. Note that this figure is considerably lower than for highly productive islands, where a mean effort of 543 ± 341 (95% CL) person‐days per 1,000 ha of island over 5.2 ± 1.6 years was required to remove cats and validate success (Parkes, Fisher, Robinson, & Aguirre‐Muñoz, 2014) and also less than the figure of <US$70 per ha reported for the large‐scale cat eradication from the 63,000‐ha Dirk Hartog Island (Algar et al., 2019). Island eradications incur extra expenses of mobilizing to the island and do not benefit from the “one‐way” fence that reduces effort required for eradications from fenced sanctuaries where cats and foxes can climb out.

Foxes and especially cats will occasionally breach even well‐constructed fences; hence, continued exclusion requires frequent monitoring and infrequent incursion management, costing in the vicinity of $40,000 per year, either through active hunting or targeting transgressors with automated control devices (Moseby, McGregor, & Read, 2020). Total 20‐year operating costs for a 10,000‐ha feral predator‐free sanctuary are therefore expected to be approximately $1.45 million.

At least 20‐km buffers (total 250,000 ha) are required around core unfenced fox‐free sanctuaries protected by baiting. Annual costs for fox baiting 720,000 ha of Flinders, Gawler, and Olary Ranges in SA (Bounceback) are estimated at $155,000 (T. Mooney personal communication), which equates to $54,000 per annum for broadscale fox baiting to protect a 10,000‐ha refuge, including buffer. This cost is just under half the costs for labor alone of largely ground‐based fox control in 2003 that cost $5,300,000 in labor over 107,000 km^2^ in Australia (Reddiex et al., 2006). Although aerial fox baiting is not permitted in all jurisdictions and is inconsistent with the safeguarding of dingoes, which can help suppress fox and cat populations, an aerial fox‐baited sanctuary would cost approximately $54,000 per annum, or $1.1 million over 20 years. Baiting, the most widespread tool for managing feral cats, is estimated to cost $1.5–$2 million to treat 1 million hectares (Commonwealth of Australia, 2015). However, unlike fox baiting which has proven to sustainably suppress fox numbers, baiting has yet to provide sustained control of free‐ranging feral cats (Johnston et al., 2013; Moseby et al., 2011). Hence, we do not consider baiting alone has been demonstrated to provide a reliable long‐term refuge from feral cat predation.

#### 4.3.3 Rabbits (20‐year cost for 10,000 ha ca $1,270,000)

Rabbit exclusion from landscape‐scale sanctuaries is typically an adjunct to feral predator exclusion, although cat‐ and fox‐proof netting is larger diameter and cheaper than rabbit‐proof fencing. Additional costs of 30 mm aperture rabbit netting for the bottom panel of exclusion fencing amount to an additional establishment cost of ca $1,300 per km, (R. Pedler personal communication) indicating an additional cost of $120,000 to reinforce a 10,000‐ha sanctuary with rabbit‐proof netting. However, with the exception of islands, eradication of high‐density rabbit populations is rarely feasible for areas larger than 2,500 ha (Read, Moseby, Briffa, Kilpatrick, & Freeman, 2011) suggesting that a 10,000‐ha rabbit‐proof sanctuary would need to be subdivided into 4 sections with a further 20 km of fencing, increasing the upfront capital costs to $360,000. Experience suggests that an average of 18 days per km^2^ using two workers is required to remove rabbits, once their numbers have been reduced by biological control and poisoning (derived from Read et al., 2011). Based on monthly personnel and logistics costs of $7,000 per person, we estimate eradication costs for a 2500‐ha paddock to be approximately $210,000, which equates to $840,000 for an entire 10,000‐ha sanctuary. Although the logistics of rabbit control were vastly different compared with fenced arid habitats, the eradication of rabbits and rodents from 12,875‐ha Macquarie Island from 2007 to 2014 costs approximately $19 million (PAWS, 2014). Note that predation by perenties (*Varanus giganteus*) significantly reduced the effort required to eradicate rabbits from the 100‐ha Donald's Well Warru Pintji in far northern South Australia, delivering considerable cost savings. Annual rabbit incursion monitoring and fence monitoring and repair costs for a 10,000‐ha sanctuary would be conducted concurrently with feral predator management at no extra costs. However, removal of rabbits from refuges occupied by other burrowing wildlife (e.g., bilbies and boodies) is challenging and can be very time‐consuming (J. L. Read, personal observation) and should be budgeted at approximately $5,000 per year. Total estimated 20‐year costs for creating and maintaining rabbit‐free status within an existing feral predator sanctuary in prime rabbit country would therefore amount to $1,150,000.

#### 4.3.4 Fire management (20‐year cost for 10,000 ha ca $418,000)

The cost of small‐scale, targeted fire management actions, such as protecting threatened species or infrastructure confined to a small area, is greater ($2.09 per ha for 5,270‐ha area, Table 3) than broadscale application of fire with aerial incendiaries and more general conservation aims ($0.19–0.60 per ha for 70,872–300,000 ha, Table 3). Due to their relatively small area and need to protect important assets, sanctuaries are likely to require targeted and strategically timed fire management over extended periods with an annual expenditure of approximately $2.09 per ha per year, or around $419,000 over 20 years.

**TABLE 3:** Case studies indicating costs of fire management

Reserve	Area managed using fire (ha)	Methods	Cost per ha	Projected cost for 10,000 ha over 20 years
Anangu Pitjantjatjara Yankunytjatjara Lands (includes New Well and Wamitjara), SA	5,270	Vehicle‐based—intensive and targeted approach	$2.09	$418,785
Ethabuka Reserve, Qld	70,872	Helicopter and incendiaries plus vehicle‐based	$0.60	$119,463
Central Land Council 600 mm+	300,000	Vehicle rangers plus some helicopter incendiaries	$0.19	$38,000

Note

Area of management determined by a 1‐km buffer around flight paths or vehicle tracks. Calculations based on once‐off fire management operations since these consumable costs are required for each burn.

The cost of fire management is dependent on methods, objectives, access, remoteness, fire history, and available resources. The three fire management case studies presented (Table 3) are from landscapes where buffel is either absent or at very low densities. Buffel increases the frequency and intensity of fire due to its prolific vegetative growth and rapid postfire recovery (Miller et al., 2010; Schlesinger et al., 2013) fueled by its deep root system. Using fire as a management tool in buffel‐invaded areas is problematic because rapid simultaneous curing throughout infestations makes fire difficult to control, and damage caused by fire to native vegetation is increased due to the higher fuel loads. Sanctuaries containing buffel will require greater investment in fire management, including selective herbicide application in order to stagger curing and exert greater control over prescribed burns. Unlike many native grasses that are largely removed by termites once dry, buffel provides high fuel loads for many years after it establishes. If prescribed burning is used as a management tool in areas with only a light infestation of buffel, it is imperative that the frequency of burning matches maturation of the native grasses as frequent burning can favor fast‐growing invaders like buffel and thereby promote further invasion (Alba, Skalova, McGregor, D'antonio, & Pysek, 2015; D'Antonio & Vitousek, 1992; Firn, House, & Buckley, 2010).

#### 4.3.5 Buffel (20‐year cost for 10,000 ha $100,280,000)

The best documented long‐term buffel eradication case study, at the Alice Springs Desert Park, costs between $50/ha/year in dry years and $10,000/ha/year in wet years, with an average of $5500/ha/year over 11 years (Gary Dinham in Friedel et al., 2008, Table 4). Whereas vertebrate pests can be largely prevented from reinvading sanctuaries, buffel seeds remain viable in the soil for many years postremoval of mature plants, are readily transported on machinery, livestock, and by wind and water, and can readily reinvade or colonize new areas. Hence, in addition to an ungulate‐proof fence, operating costs for monitoring, labor, herbicides, and equipment will be ongoing whenever buffel is present in the surrounding landscape.

**TABLE 4:** Costs of buffel control

Location/treatment	Area (ha)	Max annual cost/ha	Min annual cost/ha	Mean annual cost/ha	Source
Desert Park Alice Springs (NT)/slashing, spot spray, hand pulling		$10,000	$50	$5,500	G. Dinham in Friedel et al. (2009)
AWNRM EW rail corridor	76	$789	$7.20		T. Bowman pers. comm.
Mambray Creek (SA)/aerial granular	30			$604	T. Bowman pers. comm.
New Well (SA)/aerial granular	86			$348	APY Land Management pers comm.
Roxby Downs (SA)/hand pull, spot spray	30			$431	R. Pedler, C. Lynch. K. Solly pers. comm.
West MacDonnell Ranges (NT)/slashing, spot spraying	2	$3,848	0	$1,832	Schlesinger et al. (2020)

Note

NB With the exception of the Desert Park and West MacDonnell Ranges trials, other techniques have not proven effective long‐term and costs for sustained eradication are likely to be considerably higher than these treatment costs alone.

Abbreviations: AWNRM EW Rail, Alinytjara Wilurara Natural Resource Management Board East West railway; NT, Northern Territory; SA, South Australia.

New residual chemicals that may control buffel while minimizing effects on native grasses and other vegetation, and aerial application have the potential to decrease costs and increase long‐term efficacy of control. Evaluating and perfecting the efficacy of these herbicides, including their interaction with fire and flood and recovery of native vegetation, could revolutionize land managers’ capacity to exclude buffel from designated sanctuaries and promote regeneration and restoration of native flora and fauna species (Biosecurity SA, 2019). If residual herbicides prove effective, aerial spreading of either liquid or granular herbicide could protect landscape‐scale buffel sanctuaries for approximately 10% of the costs of ground‐based spraying, although systematic on‐ground surveillance, monitoring, and possible re‐application will be an ongoing requirement after an initial knockdown phase. Pilot trials of aerial application conducted at New Well, APY Lands, suggest costs of $348 per hectare for granular herbicide dispensed by helicopter over 86 hectares in a remote part of central Australia. Cost of aerial application trials at Mambray Creek includes a breakdown of $450 per ha for granular herbicide plus $20 per ha spreading costs and $1,050 ferrying costs (T. Bowman personal communication, Table 4). Therefore, in the initial phase at least, aerial application costs are on average 9% of the average $5500/ha/year estimated for ground‐based control (Table 4). On a broader scale and at an invading buffel front, it is unlikely that the entire sanctuary will require treatment, which will save on per ha costs. However, the efficacies and off‐target impact of aerial treatments have yet to be determined and it is likely that ground‐based monitoring and treatment will be required to supplement aerial management, and this could offset these savings. Furthermore, given that re‐establishment of native vegetation is important for sanctuary values and restricting invasive plant recolonization, additional revegetation costs may need to be budgeted for when calculating the cost of buffel management.

Even though the most reliable costings for sustained buffel suppression suggest rates of $5,500 ha/year on average (but with very high variability among years) using ground‐based techniques that reflect typical buffel management plans, we have elected to use the much discounted figure of $500/ha/year to generate a conservative estimate for 20‐year protection costs on the assumption that effective aerially spread herbicide will prove to be effective and species‐specific. These operational costs are additional to the stock‐fencing required for a buffel sanctuary at costs identical to the camel exclosure above.

## 5 DISCUSSION

In arid and semi‐arid regions of Australia, the threat to biodiversity and our perceptions of threats to cultural and social values of Aboriginal peoples posed by buffel are demonstrably of a similar scale, or even surpasses the threats caused by introduced mammals and changed fire regimes (Table 5). Our conclusions build on and broaden those of Fensham et al. (2015) who considered buffel to be the most serious threat to floristic diversity and composition in a northeastern Australian savanna ecosystem used for rangeland pastoralism.

**TABLE 5:** Overview of conservation and cultural risks of five key threatening processes and forecast costs of creating and maintaining a 10,000‐ha sanctuary for these threats for 20 years

Main threatening processes in central Australia	Assessment of risk to biodiversity	Assessment of impacts to culture and values for Aboriginal people	Ranking of environmental plus cultural risk	Estimated cost to maintain a sanctuary
		Maximum potential impact to one aspect	(1 highest–5 lowest)	20 years for 10,000 ha ($AUD, thousands)
Rabbits	Medium	Minor	5	$1,270
Large exotic herbivores	Low	Major	4	$280
Inappropriate fire regimes	Medium	Moderate	3	$418
Cats and foxes	High	Major	2	$2,130
Buffel	High	Massive	1	$100,280

Non‐native grasses in general threaten the integrity of vast areas of Australia (Firn et al., 2015; van Klinken & Friedel, 2018), yet their control and the recognition of their impact represents a chronic policy failure (Downey et al., 2010; Godfree et al., 2017). Unlike other key threats that have largely realized their potential ranges and impacts, buffel continues to rapidly spread beyond the 68% of the continent previously considered suitable for its establishment (Lawson, Bryant, & Franks, 2004) as evidenced by its invasion of new habitats and climates (Hobbs, Naby, & Schutz, 2015; Tschirner, Gibbs, & Heap, 2016). Martin et al. (2015) predicted an increased risk of buffel establishment and persistence in southern Australia, and Wilson et al. (2011) forecast that climate change will further increase this risk for large areas of New South Wales and South Australia. The future interactive effects of climate change and buffel on fire in the arid zone are likely to cause major irreversible changes in ecosystems. Furthermore, unlike other key threats, buffel is not formally listed as a key threatening process and is not afforded a national control or research strategy. In the absence of a national approach that could deliver broadscale threat amelioration, management of buffel invasion is restricted to expensive and often ineffective localized control activities.

Our review suggests sanctuaries sustained free from buffel infestation are likely to cost 40–50 times more than maintaining sanctuaries from other key threats. These estimates are consistent with projected average annual costings for broadscale buffel control in excess of $30 million a year, thirty times more expensive than control of any other non‐native plant species, in the conservation estate of the Queensland Lake Eyre basin (Firn et al., 2015). The other complexity with controlling a highly competitive invasive plant species like buffel is consideration of the disturbances and environmental conditions created by control efforts (Firn, Rout, Possingham, & Buckley, 2008). Eradicating large expanses of buffel in grassy ecosystems may open space for reinvasion by buffel or other invasive plant species—creating a “weed‐shaped hole” (Buckley, Bolker, Rees, & M., 2007). For these reasons, control efforts should focus on priority locations, and the methods used and timing of application should prioritize restoration of native plant communities rather than simply removing buffel (Firn et al., 2008). Sustainable control and transformation of invasive grasses back into desirable grasslands may take many years of strategic control (Wilson & Clark, 2001).

Despite the exorbitant costs of buffel control, our modified SEICAT analyses suggest the cultural costs of buffel invasion are significant, add to, and are intertwined with the ecological impacts, and indicate that significant expenditure is warranted. To place the “Massive” permanent risks that we believe buffel poses to bush foods, medicines, and materials, and the “Major” risk to a number of other cultural values into context, Van Dam, Walden, and Begg (2002) considered the invasion by cane toads (*Rhinella marina*), a high‐profile introduced species that has invaded extensive areas in northeast Australia and is continuing to spread across the northern tropical region into western Australia, as the lower “Major” category, because although toads led to the abandonment of certain cultural practices due to the loss of totem species, the impacts were considered reversible if toads decline. By contrast, there is little prospect of buffel declining within many decades of establishment. Therefore, despite the benefits of buffel in some pastoral areas, there is a clear and urgent need for effective buffel control on conservation estates and many Aboriginal managed lands.

The creation of landscape‐scale sanctuaries affording protection to rabbit‐vulnerable flora and fauna would not have been achievable except by capitalizing on the dramatic declines in rabbit populations post‐RHDV (Read et al., 2011). The eradication of buffel in heavily invaded areas is analogous to trying to control rabbits during plagues. Due to the technical challenge and expense of long‐term buffel eradication documented in this review, we conclude that the creation of sanctuaries from buffel either requires initiation before establishment or is predicated on broadscale eradication from infested landscapes. Other studies on prioritizing the control of invasive plant species at a landscape scale (e.g., *Mimosa pigra* in the Northern Territory) have found that conserving areas that are not yet invaded is the optimal strategy and best promotes the resilience of native communities (Firn et al., 2008). Although small‐scale buffel control in heavily infested areas may have localized benefits for flora and fauna and be integral to the conservation of particular environmental or cultural assets, prioritizing buffel control at or beyond the invasion front of buffel is generally more pragmatic and will lead to more substantive positive outcomes.

Sourcing additional biological agents that affect buffel but not native grasses are likely to be challenging (Wapshere, 1990), and success is unlikely without concerted search, development, and appraisal effort. Furthermore, given that the status quo is resulting in buffel irreversibly replacing native grasses, forbs, shrubs, and even trees over vast areas, with major to massive impacts for a range of cultural values, some off‐target impact may be preferable to no buffel control. Introduction of biological agents that are also likely to affect nontarget species may have precedence where net benefits are predicted. For example, biological control was considered justifiable to minimize the catastrophic effects of purple loosestrife (*Lythrum salicara*), because entire ecological communities would be lost in the absence of such control (Malecki et al., 1993). Similarly, introduction of a non‐native decapitating fly (*Pseudacteon curvatus*) that also attacks native fire ants was, on balance, viewed as a justifiable and pragmatic approach to controlling the imported red fire ant (*Solenopsis invicta*), due to the net ecological benefits predicted from the control of the non‐native fire ant (Porter, 2000). Likewise, although prey switching caused localized extinctions of some native mammals immediately following RHDV (Moseby, Read, Gee, & Gee, 1998), the “pest‐shaped hole” left by the extirpated rabbits was essentially filled by the recovery of threatened native mammals (Pedler et al., 2016).

Our assessment is that annual national expenditure of ca $1.5 million on buffel control, management, and research is a profoundly inadequate investment compared with the considerably greater resources directed at feral camel control ($3 million per annum) and other pests that pose less environmental and cultural risks.

In light of the immediate and expanding threat and current lack of a feasible or affordable control strategy, we propose the following actions:

1. The Federal Government urgently coordinates a national inquiry to reconsider pragmatically listing buffel as a Weed of National Significance and a Key Threatening Process, in addition to the existing Threat Abatement Advice (Australian Government, 2015), which logical appraisal of the criteria and expert opinion clearly support. Such a listing should not be conditional upon compulsory control of buffel in all areas where it has been deliberately established, but would recognize the effects and potentially facilitate control in the vast areas of Australia where buffel is already, or potentially (Martin et al., 2015), the most serious environmental weed and threatens biodiversity and culture.
2. National recognition and acknowledgment of the social and cultural risks and costs that buffel imposes on Aboriginal peoples and their ability to maintain cultural practices that have endured for thousands of years. In our view, buffel might be considered a form of cultural vandalism and constitutes a significant risk to the heritage values of Australia.
3. Funding, creation, and maintenance of 10,000‐ha buffel‐free sanctuaries in a range of habitats and locations to help preserve cultural practices and biodiversity until landscape‐scale solutions are available. To enhance feasibility of long‐term control, areas at or just beyond the invading front of buffel that support threatened species, communities, or cultural sites or practices should be targeted.
4. Raising awareness of the impacts of buffel among all stakeholders (listed as a key strategy in the Threat Abatement Advice to improve the likelihood of successful abatement of the impacts of buffel grass).
5. Coordinated and strategic research into use of both fire and herbicides, application timing, and techniques to maximize cost‐effective control of buffel from key sanctuaries and invasion fronts that is aimed at promoting the return of native plants and function to degraded ecosystems.
6. Coordinated and strategic research into how to effectively manage fire in buffel grass‐affected areas to protect people and assets without further accelerating transformation of invaded ecosystems
7. Implementation of policy that assigns responsibility for the appropriate control of buffel to land managers, pastoralists, miners, freight and infrastructure corporations, natural resource management officers, and tourists alike to prevent spread and control new outbreaks before they become unmanageable. Rangers (indigenous and nonindigenous) cannot be expected to singlehandedly restrict, let alone prevent, the spread of buffel through the Australian arid and semi‐arid zones.
8. Instigation of a national monitoring tool (possibly including remote sensing and citizen science observations) that routinely tracks the spread and persistence of buffel into existing refuges and prioritizes areas for urgent action.
9. Improved understanding of how buffel effects ecological communities to inform optimal management of sanctuaries and threatened species and to inform management in already invaded areas where buffel cannot be controlled.
10. Recognition that biological tools that reduce the dry standing biomass and hence the fire risk associated with dry buffel or reduce the viability of seed are not inconsistent with sustainable pastoral use of buffel grass and could benefit conservation, culture, and pastoral productivity. This recognition is required to recast the historic aversion to consider, let alone approve, research into biological control for buffel (Stanley & Fowler, 2004) or facilitate the spread of potential control agents due to apparent competing interests of pastoralists.
11. Searching proactively for, and potentially enhancing, endemic biological agents that could limit the encroachment, viability, or persistence of buffel in landscapes where it is considered a serious threat.

## 6 CONCLUSION

The pervasiveness of buffel and severity of current and future impacts present an urgent call to action to conserve key elements of the biota and prevent the ongoing erosion of opportunities for Aboriginal people to engage with and pass on key cultural practices in inland Australia. Creation of buffel‐free refuges is as important, yet more expensive and challenging, than creating refuges from other threatening processes. We strongly advocate collaborative research into optimal buffel control, investment into the resourcing of a network of buffel‐free refuges, and continued targeted control to protect key environmental and cultural assets from the expansion of buffel. However, even with intensive and sustained local management, there is little likelihood that the significant negative impacts of buffel will diminish until one or more biological agents reduce its invasiveness or dominance. Waiting for such an agent to naturally take effect may effectively consign dozens of species to extinction, lead to significantly reduced abundance of many other species, and perhaps irreversibly erode the customs, habitability, productivity, and biodiversity of much of central Australia. The evidence and need for prioritizing and resourcing pragmatic solutions to the buffel threat could not be clearer or more urgent.

## CONFLICT OF INTEREST

No authors disclose any conflict of interest.

## AUTHOR CONTRIBUTION

**John L. Read:** Conceptualization (lead); Methodology (equal); Writing‐original draft (lead); Writing‐review & editing (equal). **Christine A. Schlesinger:** Conceptualization (supporting); Formal analysis (equal); Investigation (equal); Writing‐review & editing (equal). **Jennifer Firn:** Conceptualization (supporting); Methodology (supporting); Writing‐original draft (supporting); Writing‐review & editing (supporting). **Ellen Ryan‐Colton:** Data curation (lead); Writing‐original draft (supporting); Writing‐review & editing (equal). **A. Grice:** Writing‐original draft (supporting); Writing‐review & editing (supporting).
