# Correction to “Ranking buffel: Comparative risk and mitigation costs of key environmental and socio‐cultural threats in central Australia”

**DOI:** 10.1002/ece3.11154

**Published:** 2024-03-13

Read JL, Firn J, Grice AC, Murphy R, Ryan‐Colton E, Schlesinger CA. Ranking buffel: Comparative risk and mitigation costs of key environmental and socio‐cultural threats in central Australia. *Ecol Evol*. 2020; 10: 12745–12763. https://doi.org/10.1002/ece3.6724

On page 12746 of the Introduction, we provided a personal communication estimate of the benefits of buffel grass to the pastoral industry from a member of the industry:

“*Buffel is currently estimated to underpin approximately 44% of the $17 billion beef cattle enterprises in Australia (G. Campbell personal communication), and its eradication would be detrimental to the pastoral industry (Friedel, Grice, Marshall, & van Klinken, 2011; Grice, Friedel, Marshal, & van Klinken, 2012).”*

We wish to correct this sentence to the following:

“*We were unable to obtain published or reliable estimates of the value or number of cattle enterprises dependent upon, or utilising, buffel pastures to compare against its environmental, economic and social costs, but acknowledge buffel eradication would be detrimental to parts of the pastoral industry (Friedel, Grice, Marshall, & van Klinken, 2011; Grice, Friedel, Marshal, & van Klinken, 2012).”*

We originally used expert opinion because published estimates dated from the 1980s, etc.

It has come to our attention that the estimate made is likely unreliable, and the interpretation of “underpinning” and “beef cattle enterprises” depends upon the viewpoint of a reader, with G. Campbell referring to the entire beef industry, not just farm‐gate sales. Although not the focus of the paper, both the 44% and $17B have been challenged, and we are unable find reliable data to verify or correct them. This economic estimate is not part of our expertise, nor the topic of the paper and would be more factual and defendable to replace with the suggested text.

We apologize for this error.

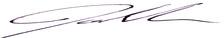

John L Read on behalf of all authors except Vale Anthony Grice.

